# A novel variant of SLC4A1 for hereditary spherocytosis in a Chinese family: a case report and systematic review

**DOI:** 10.1186/s12920-022-01399-2

**Published:** 2022-12-03

**Authors:** Jie Li, Xiaozi Wang, Na Zheng, Xiaoning Wang, Yan Liu, Liying Xue

**Affiliations:** 1 Department of Hematology, Hebei General Hospital, 348 West Heping Road, 050000 Shijiazhuang, China; 2 Laboratory of Pathology, Hebei Medical University, No. 361, Zhongshan Eastern Road, 050000 Shijiazhuang, China

**Keywords:** Hereditary spherocytosis, SLC4A1, Band 3, Case report, Systematic review

## Abstract

**Background:**

The incidence of hereditary spherocytosis (HS) is approximately 1:2000 in the western population, while it is much lower in the Chinese population. It is difficult to make a definite diagnosis due to the variable genotypic features and the lack of well-documented evidence for HS patients. Gene sequence examination is helpful for clear diagnosis.

**Case presentation:**

We presented the case of a 29-year-old male HS patient with skin yellowness, anorexia, and cholecystolithiasis as the first manifestations. Laboratory examination of the patient and his parents showed a mild reduction in hemoglobin and mean corpuscular hemoglobin concentration, increased reticulocytes, and promotion of indirect bilirubin in the patient and his father. Furthermore, small globular red blood cells with increased osmotic fragility were observed. In particular, the eosin-5’-maleimide binding test provided the strong evidence that band 3 protein was deleted in the erythrocyte membrane. Next-generation sequencing (NGS) and Sanger sequencing further demonstrated a heterozygous nonsense variant (exon16, c.G1985A: p.W662X) in SLC4A1, inherited from his father. Thus, the patient was diagnosed with HS, and then was effectively treated. After splenectomy, the anemia was relieved without any obvious unpleasant side effects.

**Conclusion:**

We report an extremely rare case of HS in China that presented with hereditary hemolytic anemia with band 3 deletion resulting from a novel variant of SLC4A1, and systematically review a large number of related literatures. This study, therefore, significantly contributes to the literature on HS.

## Background

Hereditary spherocytosis (HS) refers to a group of heterogeneous inherited anemias. In the western population, it is the most common cause of hereditary hemolytic anemia, with an estimated incidence of 1:2,000 [1,2]; however, based on clinical reports, this condition seems less common in southeast Asian and African-American populations [1]. In the Chinese population, the prevalence is approximately 1 in 100,000 people [3]. Morphologically, HS is characterized by the presence of spherocytes in peripheral blood smear, and is generally due to variants in one of the five genes (SPTA1, SPTB, ANK1, SLC4A1, and EBP42), encoding α-spectrin, β-spectrin, ankyrin, band 3 (anion exchanger 1, AE1), and protein 4.2, respectively [4]. Among these genes, ANK1 and SPTB variants are the most frequent causes of HS, followed by variants in SLC4A1 (15%) in Northern European populations [5]. However, the Asian population showed a lower rate of variant in SLC4A1, with < 13.5% in the Chinese population [6,7–11] and only 4.2% in the Indian population [12]. Although relevant studies on HS in the Chinese population have been published in the last 2 years, in some cases, it is difficult to make a definite diagnosis due to the variable genotypic and phenotypic features of HS and the lack of well-documented evidence for HS patients. Timely diagnosis and therapy will help decrease complications of biliary tract disease, such as biliary obstruction with pancreatitis, cholecystitis, and cholangitis, and contribute to improving patients’ quality of life [2,13,15]. Thus, accurate detection of known or new variant sites associated with HS is important in understanding this genetic disease.

Herein, we describe a case of HS in a 29-year-old man caused by a novel stopgain variant (c.G1985A) in SLC4A1 exon 16, inherited from his father. Notably, the patient exhibited more severe hemolytic anemia than his father and presented with splenomegaly, cholelithiasis, and kidney disease. Based on genetic screening for hereditary diseases of the hemopoietic system and immunodeficiency diseases in selected family members of the patient, we concluded that a new variant in SLC4A1 caused the phenotypic deficiency of band 3 (p.W662X) in this family, subsequently leading to the onset of HS. The patient provided written informed consent for the publication of this study, which was approved by the Ethics Committee of Hebei General Hospital, Shijiazhuang, China.

## Case presentation

**Fig. 1:**
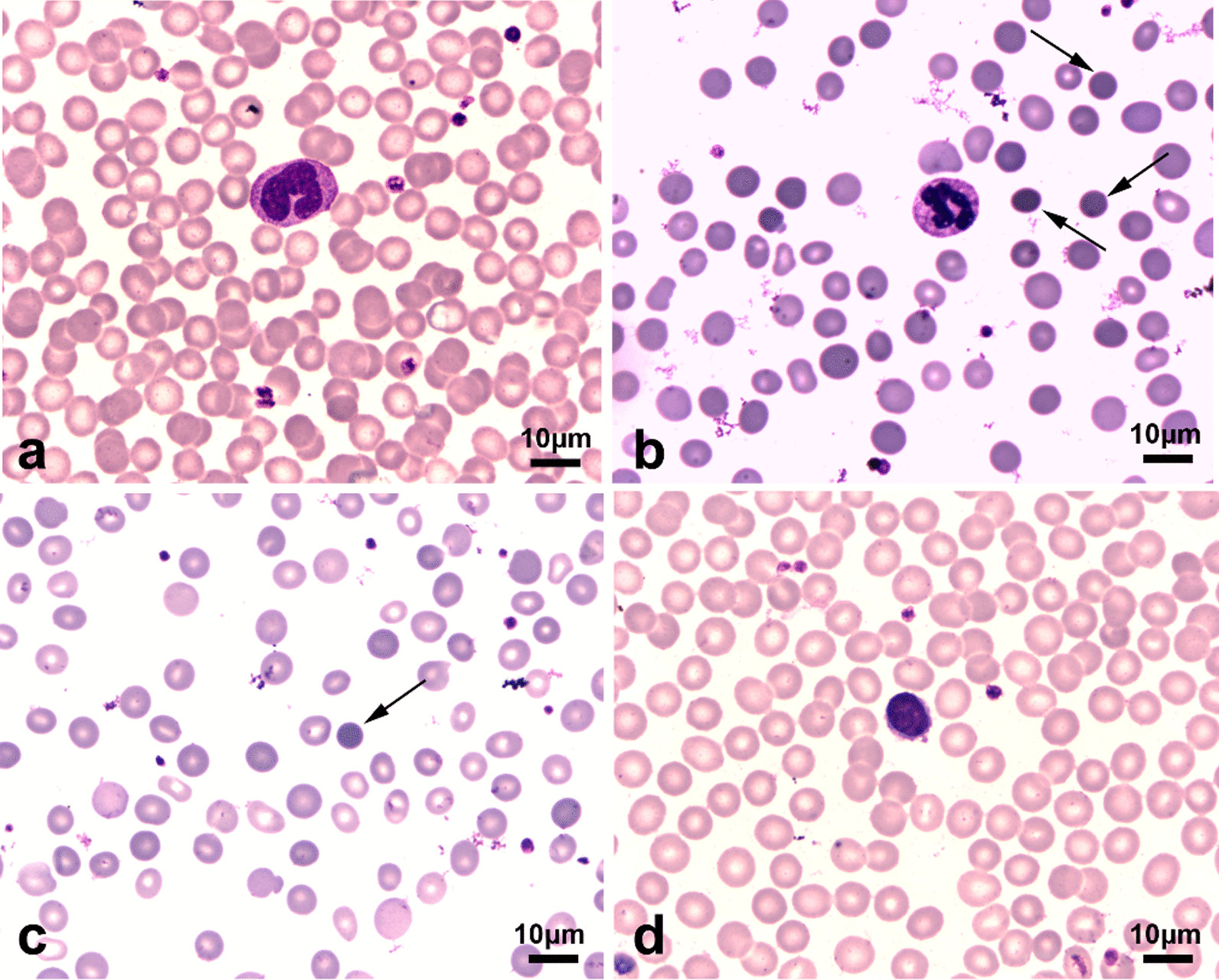
Peripheral blood smears of the patient, his parents and healthy donor. Peripheral blood smears were detected on an optical microscope (Olympus BX53, Shinjuku, Tokyo, Japan) with a cell medical image system (CMIS-2011). **a** Peripheral blood smears of the healthy donor. **b** Peripheral blood smears of the patient showed multiple spherocytes (black arrows) lacking central pallor. **c** Peripheral blood smears of his father showed many spherocytes (black arrows). **d** Peripheral blood smears of his mother. (×400)

A 29-year-old man was admitted to Hebei General Hospital (Shijiazhuang) on July 7, 2020, due to skin yellowness, anorexia, nausea, and vomiting after satiety, occasional abdominal distension, and dizziness. Four months before presenting to our hospital, the patient was initially diagnosed with cholecystolithiasis because of similar symptoms and abdominal ultrasound results (splenomegaly and bile duct neck stones) and did not receive treatment at a county hospital. The patient was subsequently admitted to our hospital with complaints of weight loss and unrelieved symptoms resulting from cholecystolithiasis. More than 10 years ago, the patient’s father showed mild anemia, splenomegaly and elevated bilirubin without a clear diagnosis and further treatment. On admission, the estimation of the complete blood count, a mild reduction in hemoglobin and mean corpuscular hemoglobin concentration was observed in the blood of the patient and his father (Table 1). Laboratory examination revealed increased number of reticulocytes and increased levels of total bilirubin and indirect bilirubin (Table 1). However, the patient showed negative results on immunofluorescence diagnosis and Coombs’ test. Peripheral blood smears for the patient and his parents showed different forms and sizes of mature red blood cell (RBC), with small globular RBC in the patient (2.4%) and his father (Fig. 1). The osmotic fragility test for the patient showed that a significant increase in RBC osmotic fragility (Table 2).

**Table 1:** Laboratory investigations

Investigations	On admission	Father	Mother
WBC (mm^3^)	3630	4660	4280
Neutrophils (%)	57.3%	51.3%	47.1%
Lymphocytes (%)	37.2%	43.8%	47.7%
Monocytes (%)	4.1%	3.6%	4.2%
Eosinophils (%)	1.1%	1.1%	0.5%
Basophils (%)	0.3%	0.2%	0.5%
Erythrocyte (mm^3^)	3,280,000	3,630,000	4,390,000
Hemoglobin (g/dl)	10.9	11.8	13.5
Platelets count (mm^3^)	189,000	187,000	227,000
Prothrombin time (seconds)	10.8	11.6	9.9
Reticulocyte (mm^3^)	466,100	410,900	52,700
Reticulocyte (%)	14.21%	11.32%	1.20%
Activated partial thromboplastin time (seconds)	25.2	28.5	27.1
Serum folate (ng/mL)	3.09	4.12	8.36
vitaminB-12 (pg/mL)	615.2	755.2	554.3
Serum ferritin (ng/mL)	693.4	525.7	32.6
Serum iron (μmol/L)	14.9	20.6	28.7
Total iron-binding capacity (μmol/L)	44.8	42.5	55.2
Transferrin (mg/dL)	196.4	183.3	262.8
Unasturated iron binding force (μmol/L)	29.9	25.3	43.7
Coombs’ test	Negative	Negative	Negative
Blood urea (mmol/L)	6.8	7.5	8.4
Serum creatinine (μmol/L)	85.2	66.3	58.7
Alanine aminotransferase (U/L)	30.6	27.8	25.5
Aspartate aminotransferase (U/L)	17.8	16.5	23.7
Total bilirubin (μmol/L)	111.5	133.0	13.8
Direct bilirubin (μmol/L)	12.3	13.0	3.8
Indirect bilirubin (μmol/L)	99.2	120.0	10.0
HBsAg	Negative	Negative	Negative
HBsAb	Negative	Negative	Negative
HCV-Ab	Negative	Negative	Negative
Anti-nuclear antibody	Negative	Negative	Negative

**Table 2:** The results of Hemolysis test

Investigations	On admission	Father	Mother	Reference values
Acidified glycerol lysis test (AGLT)	45s↓	43s↓	330s	> 290s
Osmotic fragility test (the beginning hemolysis)	0.56%↑	0.52%↑	0.45%	(0.44–0.48)%
Osmotic fragility test (the complete hemolysis)	0.48%↑	0.43%↑	0.32%	(0.28–0.36)%
Sucrose hypertonic test cold hemolysis (SHTCL)	24.3%↑	22.6%↑	10.8%	(0-16.9)%
Erythrocyte incubation osmotic fragility test	0.62%↑	0.61%↑	0.47%	(0.44–0.60)%

The down (↓) and up (↑) arrows represent abnormal value

**Fig. 2:**
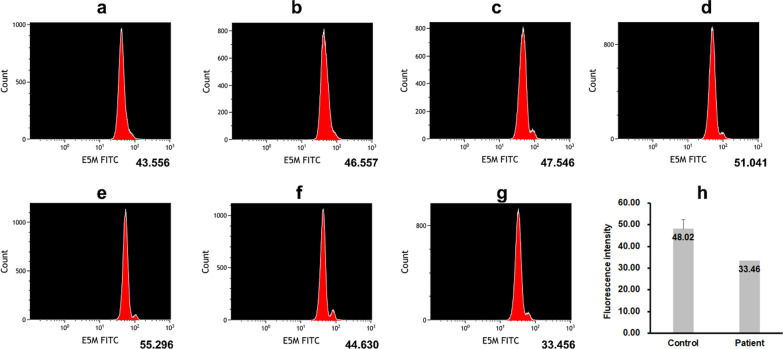
EMA binding test by flow cytometry showed decreased fluorescence of EMA-labeled RBC **g**, **h** from the patient, compared with from healthy donors **a**–**f** with a mechanical fragility index of 30.33%

**Fig. 3:**
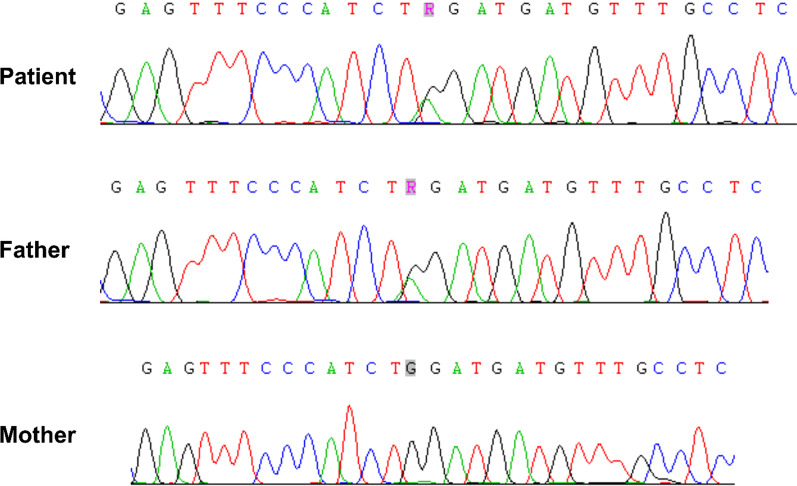
A heterozygous nonsense variant (exon16, c.G1985A: p.W662X) of SLC4A1 in the patient and his father using Sanger sequence

Furthermore, the eosin-5′-maleimide (EMA) binding test using flow cytometry (FCM) showed decreased fluorescence of EMA-labeled RBCs, with a mean fluorescence intensity of only 30.33% (Fig. 2), providing strong evidence that the band 3 protein is deleted in the RBC membrane. Based on the father’s medical history of anemia and splenomegaly, DNA from the patient and his parents were screened for nearly 700 genes related to hereditary blood and immunodeficiency diseases (SPTB, SPTA1, EPB41, EPB42, ANK1, SCL4A1, ALAS2, SFXN4, TET2, HSPA9, HBA1, MTR, MMAB, etc.) using the next-generation sequencing (NGS). The results showed a heterozygous nonsense variant (NM_000342: exon16, c.G1985A: p.W662X) in SLC4A1. Sanger sequence further demonstrated that this variant was inherited from his father, but not from his mother (Fig. 3). According to the Mutational Database, including 1000 Genomes Project, dbSNP, ClinVar, ESP6500, ExAc, Ensembl, HGMD, and UCSC, this variant has not been reported previously. Meanwhile, it was predicted to be pathogenic variant (PVS1 + PS1 + PM2) based on the American College of Medical Genetics and Genomics (ACMG) standards and guidelines.

**Fig. 4:**
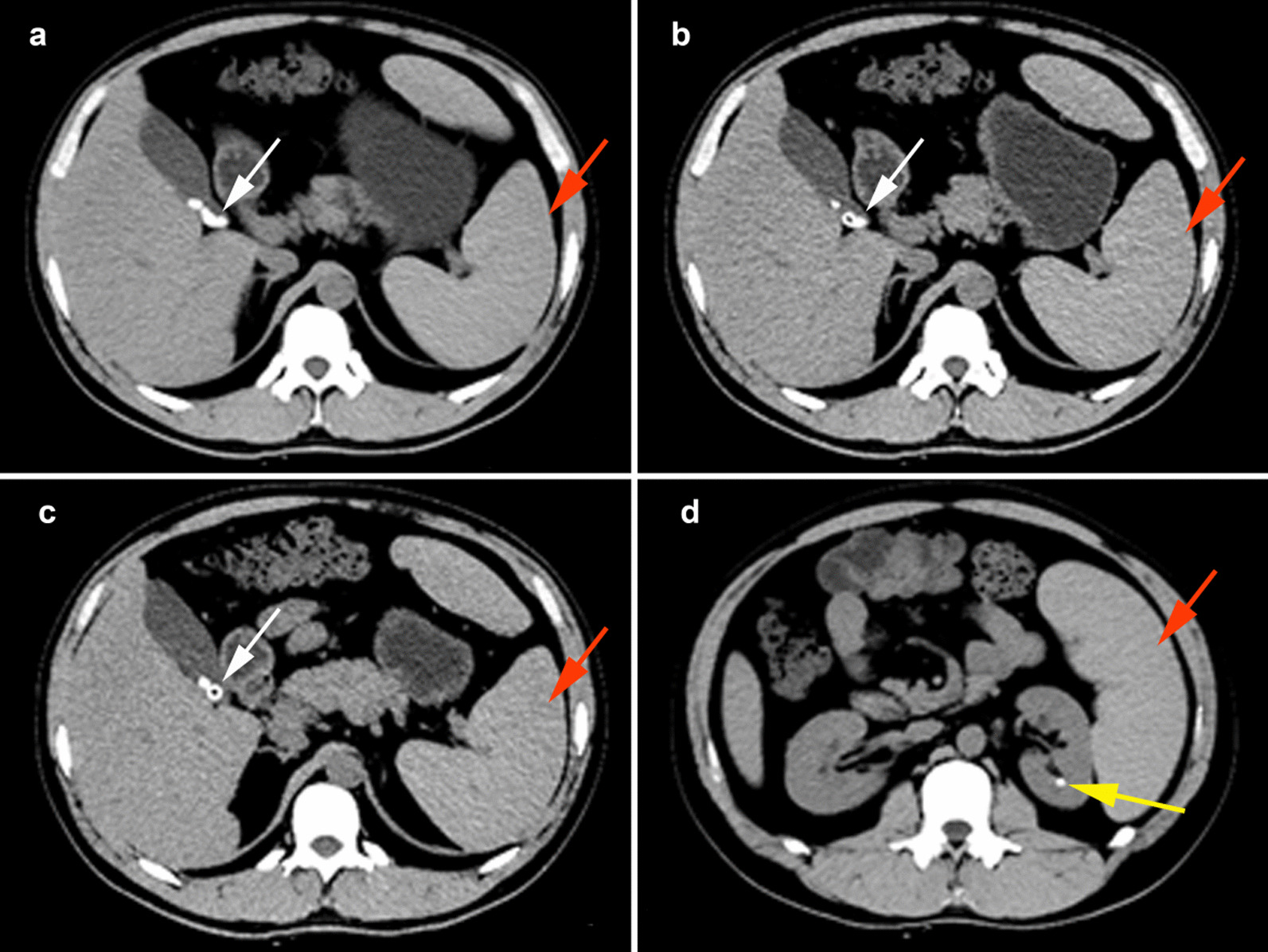
Abdominal and pelvic CT scans showed multiple gallstones (white arrows in **a**, **b** and **c**), splenomegaly (red arrows in **a**, **b**, **c** and **d**) and left renal calculi (yellow arrow in **d**)

Additionally, abdominal ultrasound showed fatty liver, hepatomegaly, multiple gallstones, splenomegaly, and splenic vein widening. Abdominal and pelvic computed tomography (CT) further demonstrated multiple gallstones, splenomegaly, and left renal calculi (Fig. 4).

Based on the prominent symptoms, laboratory results, and DNA screening, the patient was diagnosed with HS, gallbladder stone, and left kidney stone. He began treatment with folic acid and mecobalamin. After splenectomy, the anemia was relieved without any obvious unpleasant side effects. And no occurrence of anemia was observed in the follow-up period.

## Discussion and conclusion

The prevalence of HS is relatively high in North European countries but is much lower in the Asian population. A survey of hereditary hemolytic anemia in South Korea showed 71.3% of RBC membranopathies [16], while there has been no investigation on such a large number of cases due to the sporadic nature in China. Clinical data from the Changhai Hospital showed that of the hereditary hemolysis cases, 42.56% were membranopathies [17]. However, with the clinical application of gene sequencing, the number of reported cases of HS has significantly increased [18]. In the past 10 years, nearly half of the total HS cases have been reported, with 71% diagnosed at university hospitals [18]. Owing to the lack of accurate data on the incidence of and a detailed study on HS, its early diagnosis remains unclear in the Chinese population. As HS is caused by variants in different genes, its clinical manifestations vary widely, ranging from asymptomatic hemolysis to transfusion dependence. Therefore, it is difficult to obtain the correct diagnosis and to provide early treatment through traditional examinations. Sequence analysis of genetic exons contributes to early diagnosis and understanding of the characteristics of the variants.

Physiologically, in the RBC membrane, an enormous number of transporters and channels determine RBC volume and intracellular water content. There are five causative genes of HS: SPTB, SPTA1, ANK1, SLC4A1, and EPB42, which encode the erythrocyte membrane proteins β-spectrin, α-spectrin, ankyrin 1, band 3, and protein 4.2, respectively. Among these, the tetramer of spectrin forms a dense network, lining the inner surface of the lipid bilayer in the RBC membrane, while ankyrin-1 provides the main membrane binding site for the spectrin-based membrane skeleton and links β-spectrin to band-3 [2,19]. These skeleton proteins provide RBCs with deformability and undergo substantial distortion without fragmentation during microcirculation [19,20]. Therefore, protein defects caused by gene variants result in decreased deformability, increased osmotic fragility, and premature destruction in the spleen. It has been reported that 75% of HS cases are associated with dominant inheritance and 25% are associated with non-dominant and recessive inheritance [5,21,22, 23]. In Northern European populations, variants in ANK1 (50–60%) are the most frequent cause of HS, followed by variants in the SPTB or SPTA1 gene (20%) and in the SLC4A1 gene (15%) [5]. In 25 Korean patients, variants in ANK1 (52%) or SPTB (48%) were genetically reported to be the cause of HS [24], while heterozygous variants in ANK1 were found in 31% of Japanese HS patients [25]. A study of 73 Indian families (113 patients) with HS found variants in ANK1 (53.2%), SPTB (36.2%), and SLC4A1 (4.2%) [12]. The five most recent studies reported in the Chinese population showed incidences of 44.7–66.7% for ANK1 variant, 33.3–45.7% for SPTB variant, and < 13.5% for SLC4A1 variant [6,7–11]. Compared to European countries, Asian countries show a lower rate of variants in SLC4A1, which are predominantly inherited.

In the present study, the patient presented with typical manifestations, including jaundice, anorexia, occasional abdominal distension, and dizziness. Laboratory examination showed an increased number of reticulocytes and increased levels of total bilirubin and indirect bilirubin, suggestive of RBC damage, compensatory erythrocytosis, and hemolysis. However, the negative results of immunofluorescence diagnosis and Coombs' test excluded the possibility of paroxysmal nocturnal hemoglobinuria and autoimmune hemolytic anemia. Peripheral blood smears showed small globular RBC, and osmotic fragility tests showed increased osmotic fragility. Abdominal and pelvic CT further demonstrated multiple gallstones, splenomegaly, and left renal calculi. Subsequently, EMA-FCM provided strong evidence that the band 3 protein was deleted in the erythrocyte membrane. Based on these results, the patient was diagnosed with HS. To clarify HS diagnosis and genetic variant, WES for approximately 700 genes associated with hereditary diseases of the blood and immune system was performed on samples from the patient and his father. The results demonstrated a heterozygous stopgain variant in SLC4A1 exon16 (c.G1985A; p.W662X).

SLC4A1, consisting of 20 exons, encodes a 911 amino acid protein, Band-3 (referred to as NM_000342, NP_000333.1). Erythrocyte band 3 is a major membrane protein, with 1.2 million copies per cell. Functionally, it includes two major domains: (1) an N-terminal cytosolic domain (cdAE1), providing attachment sites for the skeleton (ankyrin 1, protein 4.1, adducin2, and protein 4.2), glycolytic enzymes, and deoxyhemoglobin [26,27], and (2) a C-terminal membrane domain (mdAE1), which forms the anion-exchange channel and aids carbon dioxide transport through the exchange of chloride and bicarbonate ions [19,28,29]. The mdAE1 of Band 3 consists of 14 transmembrane (TM) segments with the N- and C-termini facing the cytosol [30]. Among these segments, a core domain (TM1-4 and TM 8–11) provides anion-binding sites and a gate domain (TM5-7 and TM12-14) contains lysine residues crosslinked by some organic anions [30]. The membrane domain of Band 3, responsible for the transport function, is completely functional in the absence of the cytosolic domain [31].

**Fig. 5:**
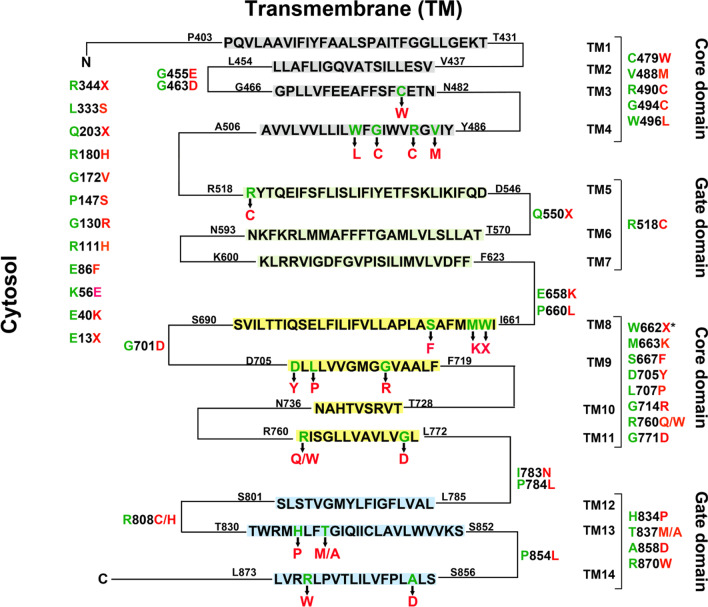
Amino acid variants in Band 3 summarized following extensive review of the literature. *presents the novel variant in this study

Variants in SLC4A1 have been linked to four human diseases, including HS, Southeast Asian ovalocytosis (SAO), and hereditary stomatocytosis (HSt) [32], distal renal tubular acidosis (dRTA) [33,34, 35]. SAO mutates frequently via the deletion of nine amino acids (Ala400-Ala408) on the cytosolic boundary region of TM1 [30], while many HSt variants cluster in or around TM10 on the cytoplasmic half of the core domain. For HS, variants in SLC4A1 are thought to occur throughout the sequence, including both the membrane and cytosolic domains. Although approximately one-third of the variants were reported to likely affect the processing of SLC4A1 pre-mRNA [1], variants in HS are common in the Band 3 membrane domain. Following extensive review of the literature, we summarized most of the amino acid variants in Band 3 (Fig. 5) [1,6,9,36,37–54]. To date, approximately 70% of the variants seem to occur in the membrane domain, more frequently than in the core domain.

In the case presented here, NGS results demonstrated a heterozygous variant (exon16, c.G1985A: p.W662X) in Band 3. Trp662, located near the beginning of TM8 (I661-S690), faces the terminal region of TM3. It has been reported that tryptophan residues in Band 3 play key roles in energy transfer. For example, Trp848, located on the extracellular end of TM13, may be the predominant tryptophan residue responsible for energy transfer [20]. Variant of Trp492 or Trp496 within TM4 and in close contact with the N-terminal region of TM8 causes Band 3 to misfold. Meanwhile, the variant of Trp648, Trp662, or Trp723 to Ala has the same effect. In this study, the variant (c.1985G > A) in SLC4A1 (inherited from the father) caused the conversion of TGG (Trp) to a stop codon TAG, resulting in the loss of Band 3. This compromised the stability of the cytoskeleton in the RBC membrane and induced the onset of HS in the patient.

In summary, we report an extremely rare case of HS in a Chinese population that presented hereditary hemolytic anemia with the deletion of band 3 resulting from a novel variant (exon16, c.G1985A: p.W662X). Because of the lack of a registration system for HS in China, the exact rate of its incidence and genotypic and phenotypic features of HS patients carrying variants in SLC4A1 remain unknown. Identifying potential genetic causes is helpful in understanding the correlation between genotype and phenotype. This study thus makes a significant contribution to the literature on HS.

## Data Availability

The NGS data generated and/or analyzed during this study are available in the NCBI Sequence Read Archive (SRA) repository (Accession Number: SRR20046343, SRR20046344, SRR20046345).

## Abbreviations

HS: Hereditary spherocytosis
AE1: Anion exchanger 1
RBC: Red blood cell
FCM: Flow cytometry
EMA: Eosin-5′-maleimide
WES: Whole exome sequencing
CT: Computed tomography
TM: Transmembrane
SAO: Southeast Asian ovalocytosis
HSt: Hereditary stomatocytosis
dRTA: Distal renal tubular acidosis
WBC: White blood cell
AGLT: Acidified glycerol lysis test
SHTCL: Sucrose hypertonic test cold hemolysis
NGS: Next-generation sequencing
